# Chronic low-dose-rate ionising radiation affects the hippocampal phosphoproteome in the ApoE^−/−^ Alzheimer's mouse model

**DOI:** 10.18632/oncotarget.12376

**Published:** 2016-09-30

**Authors:** Stefan J. Kempf, Dirk Janik, Zarko Barjaktarovic, Ignacia Braga-Tanaka, Satoshi Tanaka, Frauke Neff, Anna Saran, Martin R. Larsen, Soile Tapio

**Affiliations:** ^1^ Institute of Radiation Biology, Helmholtz Zentrum München, German Research Center for Environmental Health GmbH, Neuherberg, Germany; ^2^ Department of Biochemistry and Molecular Biology, University of Southern Denmark, Odense, Denmark; ^3^ Institute of Pathology, Helmholtz Zentrum München, German Research Center for Environmental Health GmbH, Neuherberg, Germany; ^4^ Institute for Environmental Sciences, Rokkasho, Japan; ^5^ Laboratory of Biomedical Technologies, Agenzia Nazionale per le Nuove Tecnologie, l'Energia e lo Sviluppo Economico Sostenibile (ENEA), Rome, Italy

**Keywords:** synaptic plasticity, hippocampus, dendritic spine, synapse, phosphoproteomics

## Abstract

Accruing data indicate that radiation-induced consequences resemble pathologies of neurodegenerative diseases such as Alzheimer's. The aim of this study was to elucidate the effect on hippocampus of chronic low-dose-rate radiation exposure (1 mGy/day or 20 mGy/day) given over 300 days with cumulative doses of 0.3 Gy and 6.0 Gy, respectively. ApoE deficient mutant C57Bl/6 mouse was used as an Alzheimer's model. Using mass spectrometry, a marked alteration in the phosphoproteome was found at both dose rates. The radiation-induced changes in the phosphoproteome were associated with the control of synaptic plasticity, calcium-dependent signalling and brain metabolism. An inhibition of CREB signalling was found at both dose rates whereas Rac1-Cofilin signalling was found activated only at the lower dose rate. Similarly, the reduction in the number of activated microglia in the molecular layer of hippocampus that paralleled with reduced levels of TNFα expression and lipid peroxidation was significant only at the lower dose rate. Adult neurogenesis, investigated by Ki67, GFAP and NeuN staining, and cell death (activated caspase-3) were not influenced at any dose or dose rate. This study shows that several molecular targets induced by chronic low-dose-rate radiation overlap with those of Alzheimer's pathology. It may suggest that ionising radiation functions as a contributing risk factor to this neurodegenerative disease.

## INTRODUCTION

Alzheimer's disease (AD) is a chronic neuro- degenerative disease with a progressive pattern of cognitive impairment. Characteristic features of AD include the formation of amyloid plaques originating from amyloid precursor protein (APP) and neurofibrillary tangles containing hyperphosphorylated tau protein in the brain. Already now it is the leading cause for dementia in the elderly. As the global prevalence of AD is supposed to increase dramatically in the following decades up to 80 million patients by 2040 [1], it is crucial to elucidate potential contributing factors and their role in the molecular aetiology of AD. Ionising radiation could be one such a factor [2].

Large number of people of all age groups are increasingly exposed to ionising radiation from various sources [2]. Many individuals receive chronic occupational exposure related to nuclear technologies or airline travel. The use of medical diagnostics and therapeutic radiology has increased rapidly [3]. For example more than 62 million CT scans per year are currently carried out in the United States [4]. Approximately one third of all diagnostic CT examinations are scans of the head region [4]. The absorbed tissue doses range from 10–100 mGy for a single CT examination [5] but as CT scans are frequently repeated the cumulative doses will inevitably increase for many patient groups.

Recent data suggest that even relatively low radiation doses, similar to those received from a few CT scans, could trigger molecular changes associated with cognitive dysfunction, resembling that seen in normal aging and AD [6]. A total body dose of 0.5 Gy administered to neonatal NMRI mice has been shown to result in long-term cognitive dysfunction and enhanced level of total tau protein (Mapt), a marker of AD pathology, in the adult mouse brain [7]. Total body irradiation of 8-week-old C57Bl6/J mice (0.1 Gy) induced early transcriptional response of several AD-related genes in hippocampus but no late AD-like pathogenesis or memory impairment [8]. In contrast, APP/PS1 mice, a model of AD, had permanent decreased cognitive abilities measured by contextual fear conditioning and novel object recognition tests 6 months after exposure to 0.1 or 1.0 Gy (56)Fe radiation [9]. Furthermore, an acceleration of amyloid β (Aβ) plaque pathology was observed in male mice [9].

Studies in human and animals indicate that synapses are affected at an early stage in AD neurodegeneration [10]. The hippocampus, the brain area critical for learning and memory, is especially vulnerable to damage at early stages of AD [11]. Adult neurogenesis occurs almost exclusively in the dentate gyrus of the hippocampus [12]. Several studies have shown that adult neurogenesis is inhibited by high radiation doses in radiotherapy patients [13] and in mice [14, 15], resembling the neurodegeneration seen in the AD pathophysiology [16, 17]. Neurogenesis in the hippocampus relies on the ApoE function [18].

The aim of this study was to elucidate molecular alterations in the murine hippocampus induced by chronic low-dose-rate ionising radiation. ApoE deficient C57BL/6 mice were used as an AD model; the APOE genotype is known as the major genetic risk factor for AD in human supressing synaptic plasticity, lipid transport and metabolism [19]. The mice were total body exposed to cumulative doses of 0.3 Gy or 6.0 Gy given at low dose rates of 1 mGy/day or 20 mGy/day, respectively, during 300 days. As both phosphorylation and N-linked glycosylation of proteins can influence neural cell adhesion, axonal targeting and neuronal transmission and thereby modulate synaptic plasticity we aimed to study especially these protein modifications in this study. We show here that both dose rates are capable of inducing molecular features, particularly on the phosphoproteome level, that are reminiscent of those found in the AD neuropathology.

## RESULTS

### Chronic low-dose radiation targets protein phosphorylation

8-week old female C57BL/6 ApoE^−/−^ mice were chronically irradiated with dose rates of 1 mGy/day or 20 mGy/day over 300 days with cumulative doses of 0.3 Gy or 6.0 Gy, respectively. A quantitative proteome analysis of unmodified proteins, phosphoproteins and N-linked sialylated glycoproteins was performed from the complete hippocampus.

**Figure 1:**
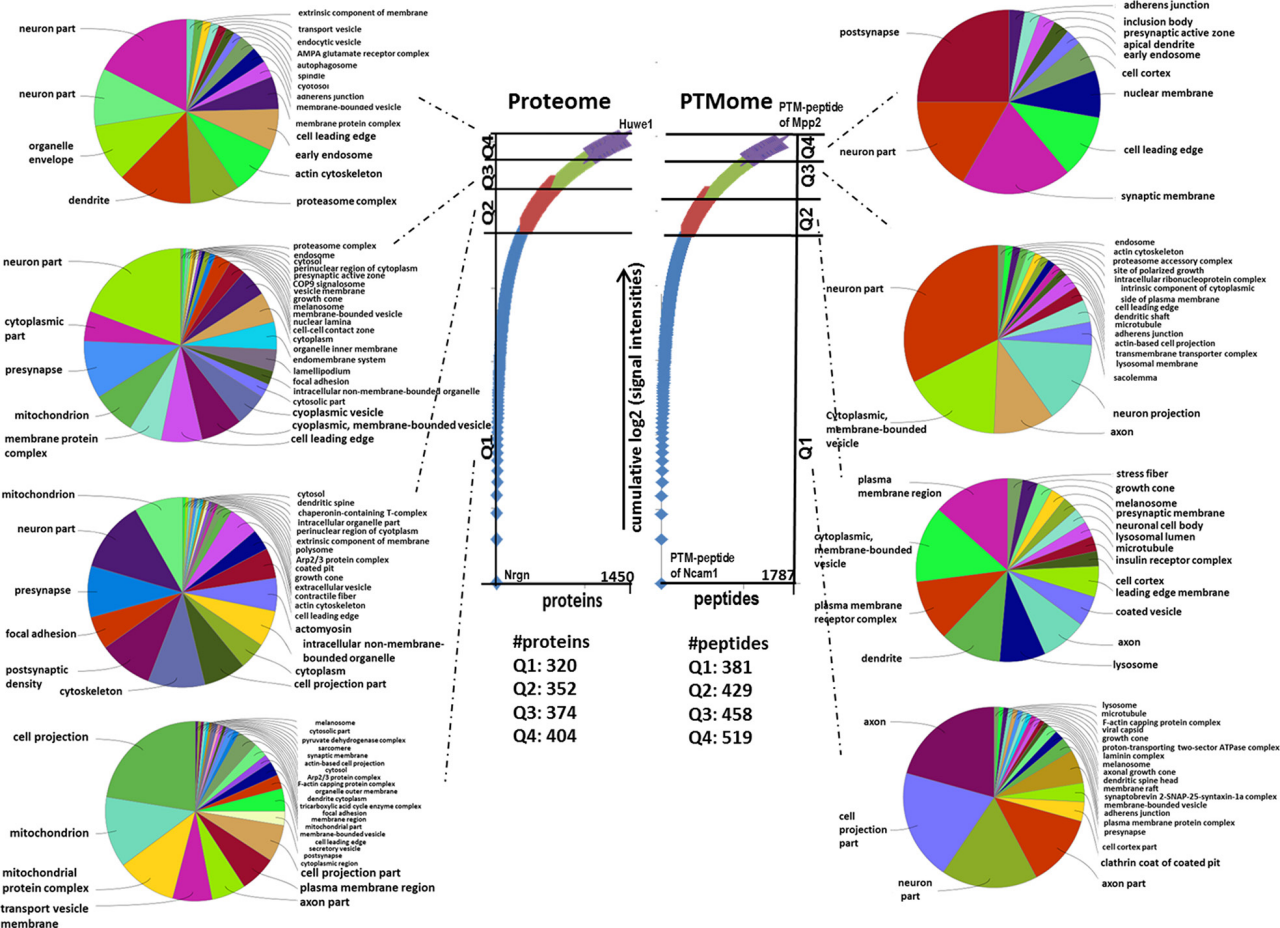
Gene Ontology “cellular compartment” of unmodified proteins and PTM peptides belonging to different quartiles. The numbers of proteins and PTM-peptides grouped into quartile Q1 (up to 25 %), Q2 (25–50 %), Q3 (50–75 %) and Q4 (75–100 %) based on their cumulative log2-signal intensities ranging from the highest to the lowest abundant are shown. This was done following Sharma et al. [57]. The hits per quartile were exported into ClueGO gene ontology software to identify enriched “Cellular Compartment” with a *q*-value ≤ 0.05 (*p*-value ≤ 0.05, corrected with Benjamini & Hochberg).

A nearly equal distribution of proteins within the proteome and post-translational modifications (PTMs) within the quartiles Q was observed (Figure 1). The quantified unmodified and modified proteins were found localised in neurons, cell projections, axons, mitochondria and synaptic membranes in an unbiased manner from high abundant (Q1) to low abundant proteins (Q4) (Figure 1).

**Figure 2:**
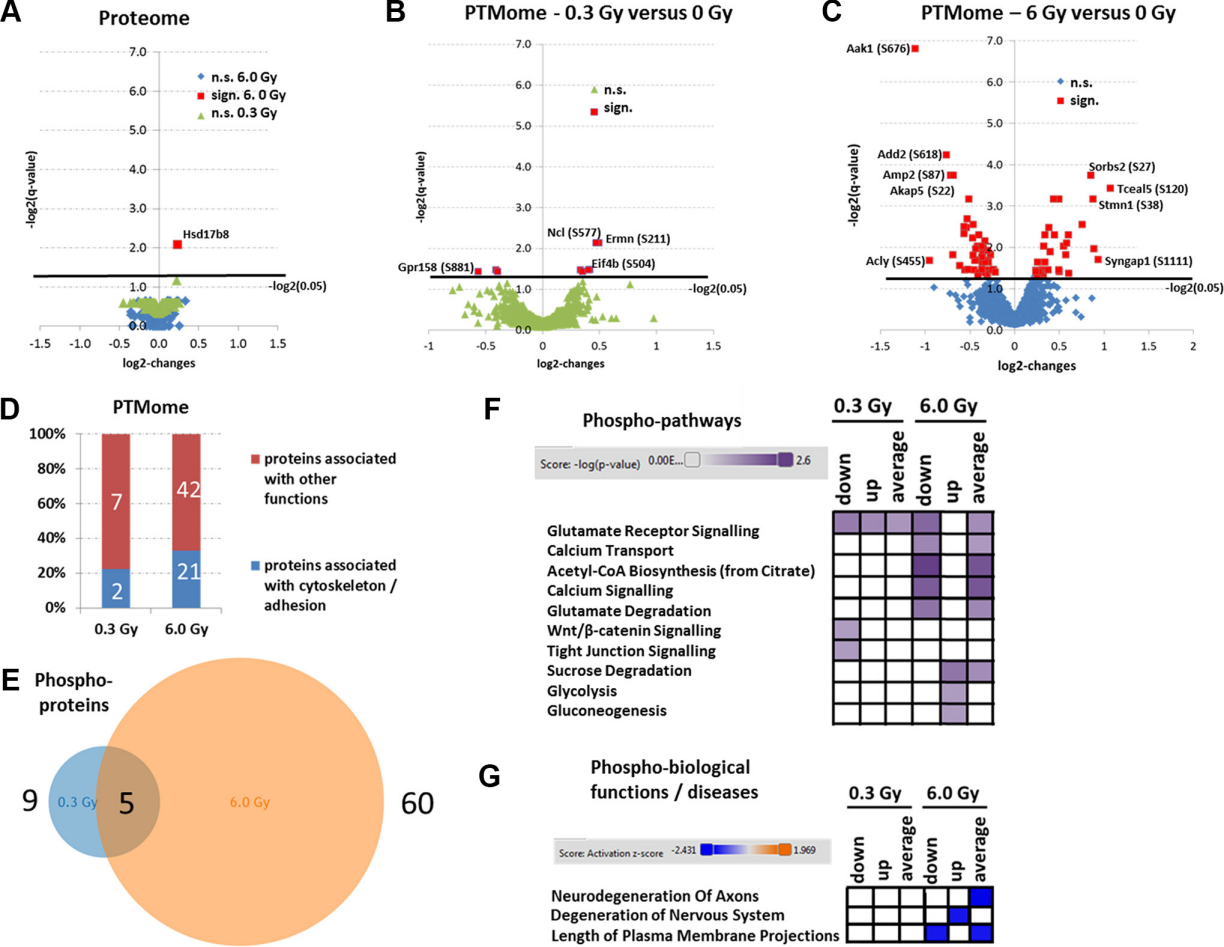
Analysis of mass spectrometry-based proteomics and signalling pathways of deregulated phosphoproteins. Panels **A**, **B** and **C** show the volcano blots (log2-changes versus –log2 (*q*-values)) of statistically quantifiable proteins (A) and PTMs (B and C); n.s., not significant; sign., significant. Panel **D** shows the percentage of PTM-proteins that belong to cytoskeleton or adhesion proteins at 0.3 Gy and 6.0 Gy. The calculation is based on the protein affiliations into sub-protein classes obtained from the PANTHER software (PANTHER protein class) and information from UniProt (Supplementary Table S2; PANTHER protein classes are highlighted in yellow) involving cytoskeleton-associated processes and cell adhesion-associated processes. Panel **E** shows the total number of deregulated phosphoproteins at 0.3 Gy (9) versus 6.0 Gy (60) with 5 overlapping hits. Panel **F** shows the affected signaling pathway analysis using IPA software. High colour intensity represents high significance (*p*-value). All coloured boxes have a *p*-value of ≤ 0.05; white boxes have a *p*-value of ≥ 0.05 and are not significantly altered. Panel **G** shows the biological functions and diseases associated to PTMome changes. High colour intensity represents high significance (z-score). All coloured boxes have a z-score of < 2.0 indicating significant inhibition of the hit; white boxes have a z-score > 2.0 and are not significantly altered. Data for panel F and G involve only deregulated phosphoproteins. All data were independently uploaded using identified phosphoproteins and fold-changes and taking into account only the down-regulated phosphopeptides (down), up-regulated phosphopeptides (up) or all deregulated peptides (average). The fold-change per phosphoprotein was then calculated accordingly for each upload.

Importantly, only one unmodified protein was altered in expression at 6.0 Gy (Hsd17b8 - Estradiol 17-beta-dehydrogenase 8) (Figure 2A); no expression changes in unmodified proteins were noted at 0.3 Gy (Supplementary Table S1). However, this was not due to an increased total variance as the average standard deviation of all unmodified proteins used for statistical analysis was 9% (Supplementary Table S1). Moreover, the biological log2-changes of these proteins were around 0 (fold-change 1) (Figure 2A), indicating that the proteome was confidently quantified within the six biological replicates.

In contrast, we noted a dose-dependent increase in the number of significantly deregulated phosphoproteins without additional glycosylation motif (0.3 Gy/6.0 Gy: 9/60) (Figures 2B and 2C, red marking). Some significantly altered phosphoproteins overlapped between the two conditions (0.3 Gy and 6.0 Gy) (Figure 2E). Upregulations in the phosphorylation profile of neurofilament (Nefm-S769 and Nefm-S723) and eukaryotic translation initiation factor (Eif4b-S504), and downregulation in phosphorylation of G-protein coupled receptor (Gpr158-S881) were observed at both doses (Supplementary Table S2). Ermin had different direction of deregulation and distinct phophosites at the two doses.

Only three glycoproteins were significantly changed at 6.0 Gy, two of which had additionally significant changes in the phosphorylation: paralemmin-1 (Palm-T145/N149) and plasma membrane calcium-transporting ATPase 2 (Atp2b2-N1154/S1155). Only one protein, neurofascin (Nfasc-N1050), was found changed on glycosylation level at 6.0 Gy (Supplementary Table S2) whereas no significant changes on this modification were found at 0.3 Gy (Supplementary Table S2).

In spite of the large change in the PTMome at 6 Gy, no significant radiation-induced alteration in body weights, brain weights or brain-to-body weight ratios were noticed (Supplementary Figure S1).

### Chronic low-dose ionising radiation targets synaptic plasticity and cellular metabolism

Pathway analysis of significantly changed phosphoproteins showed that most pathways affected at 6.0 Gy were involved in synaptic plasticity (glutamate receptor signalling, calcium transport, calcium signalling) as well as brain metabolism (Acetyl-CoA biosynthesis, glutamate degradation, sucrose degradation, glycolysis and gluconeogenesis) (Figure 2F). In contrast, only three significantly altered signalling pathways related to synaptic plasticity were observed at 0.3 Gy (glutamate receptor signalling, Wnt/β-catenin signalling, and tight junction signalling) (Figure 2F). The pathways are shown in Supplementary Table S5. Moreover, a significant inhibition of cytoskeleton-associated synaptic neurodegenerative processes (neurodegeneration of axons, degeneration of nervous system and length of plasma membrane projections) was predicted in hippocampus at 6.0 Gy but not at 0.3 Gy (Figure 2G). GO term analysis of molecular functions showed that several PTM-proteins changed in their phosphorylation status were associated with cytoskeleton and adhesion (~ 22% at 0.3 Gy and 33% at 6.0 Gy) (Figure 2D, Supplementary Table S2; PANTHER protein classes highlighted in yellow). Key cytoskeletal proteins that are also typically altered in AD such as stathmin 1 (Stmn1), microtubule-associated protein tau (Mapt), and microtubule-associated proteins 1b and 2 (Map1b, Map2) were decreased in their phosphorylation status, particularly at the higher dose. Three proteins were changed in their N-linked sialylated glycosylation pattern (Atp2b2, Nfasc, Palm). These proteins are important in synaptic plasticity regulation: Atp2b2 in ATP hydrolysis and calcium transport, Nfasc in cell adhesion, and neurite extension and synaptogenesis, and Palm in axonal and dendritic filopodia induction, and synapse formation.

### Irradiation targets synaptic plasticity by stimulating Rac1-Cofilin and inhibiting cAMP/PKA/MAPK/CREB pathways, particularly on the phosphorylation level

To further validate synaptic signalling pathways, 84 different gene transcripts important for synaptic plasticity were quantified. Only sham-irradiated and 6.0 Gy-irradiated samples were measured as most changes in the phosphoproteome were seen at this dose (Figure 2F and 2G).

The transcriptome analysis showed similar results as the proteomics-based quantification of the unmodified proteins as only four genes (*Grm2, Klf1, Ngf and Nptx2*) out of 84 were changed in their expression status (Supplementary Table S4). However, the decreased gene expression of metabotropic glutamate receptor 2 (Grm2), and the nerve growth factor (Ngf), correlated well with the bioinformatics analysis of the phosphoproteome at 6.0 Gy (Figure 2F and 2G). Neuronal pentraxin 2 (Nptx2) that was also found decreased on the gene expression level is involved in the modification of calcium-mediated long-term plasticity of synapses (Figure 2F).

**Figure 3:**
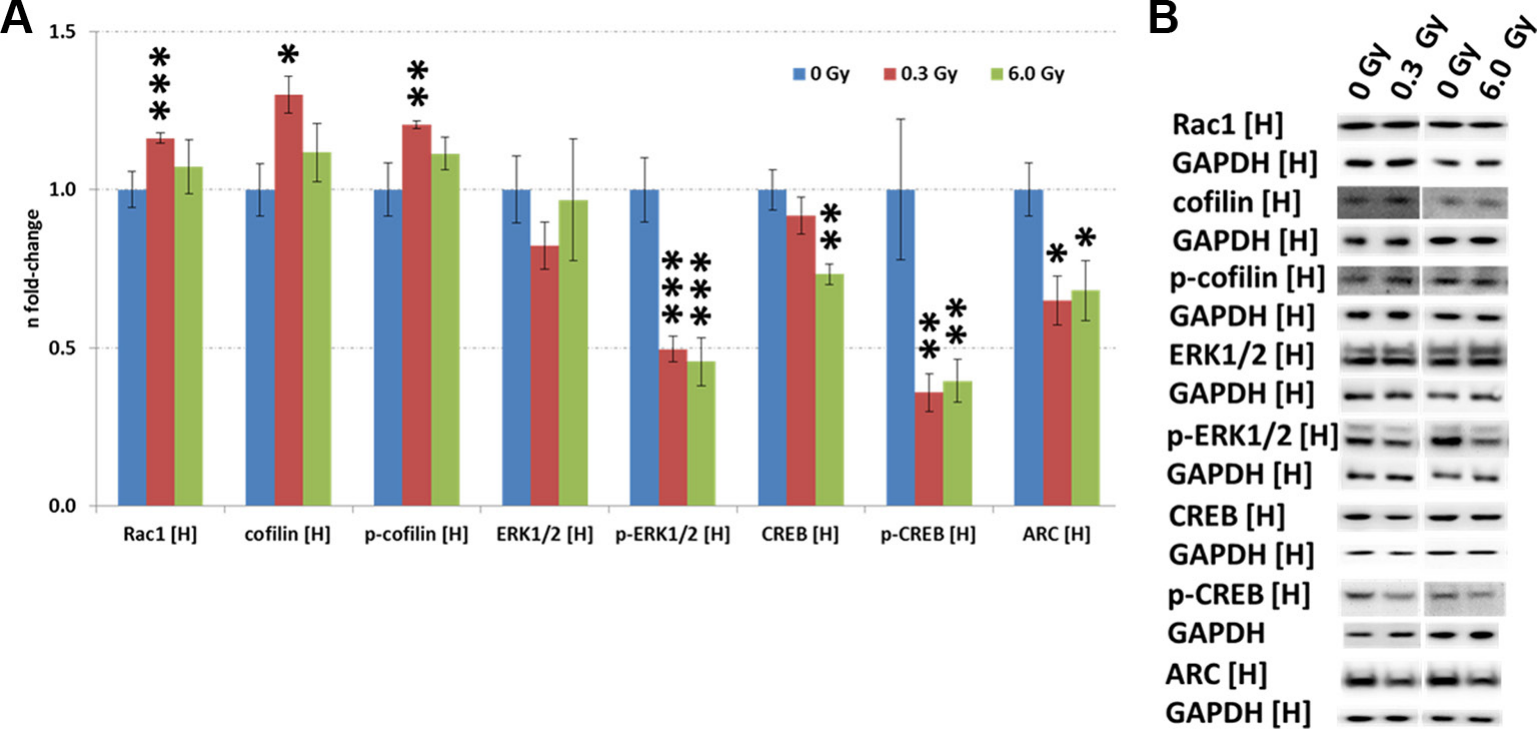
Analysis of CREB and Rac1-Cofilin signalling in the irradiated hippocampus. Panel (**A**) shows the protein levels of Rac1, cofilin, p-cofilin, ERK1/2, p-ERK1/2, CREB, p-CREB and ARC using immunoblotting. The columns represent the fold-changes with standard errors of the mean (SEM) from 6 biological replicates (*p < 0.05; **p < 0.01; ***p < 0.001 - unpaired Student's *t*-test). Panel (**B**) shows the representative images of the immunoblots. H, hippocampus.

Our previous data using acute low-dose irradiation show immediate and long-term alteration of the Rac1-Cofilin pathway [20–22]. This pathway modulates the synaptic cytoskeleton, a target predicted to be affected also in this study (Figure 2D). Indeed, increased expression of Rac1, cofilin and phospho-cofilin were found in this study but only at 0.3 Gy (Figure 3A and 3B).

CREB (cAMP response element-binding protein) is an essential transcription factor inhibited by ionising radiation [20, 23] and in AD [24]. Calcium signalling, one of the most significantly altered pathways related to synaptic plasticity in our study (Figure 2F), includes CREB signalling (Supplementary Table S5). Quantification of CREB and p-CREB on Ser133, the main phosphorylation site for protein activation, showed reduced levels of total CREB (6 Gy) and phospho-CREB (0.3 Gy and 6.0 Gy) (Figure 3A and 3B). Also the level of ARC (activity-regulated cytoskeleton-associated protein), a CREB target, was downregulated at both doses (Figure 3A and 3B). The gene expression of ARC was not significantly changed (Supplementary Table S4), nor it was identified in the proteomics analysis, presumably due its very low abundance.

**Figure 4:**
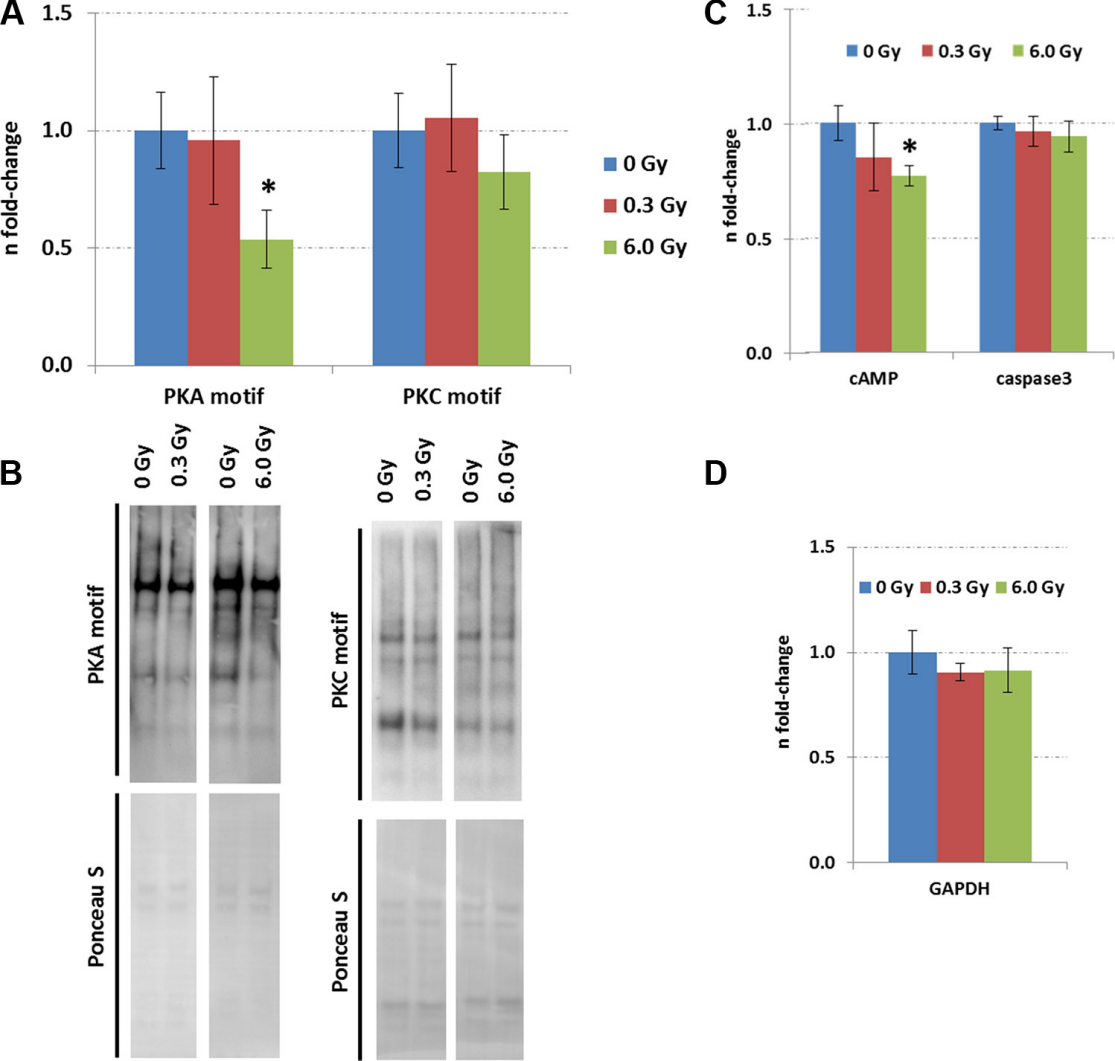
Expression of proteins with PKC and PKA motifs in the control and irradiated hippocampus. Panel (**A**) shows the data from immunoblotting of proteins with PKC and PKA motifs. The columns represent the fold-changes with standard errors of the mean (SEM) from 6 biological replicates (*p < 0.05; **p < 0.01; ***p < 0.001 - unpaired Student's *t*-test). Panel (**B**) shows representative images of the immunoblots. Panel (**C**) shows the levels of cAMP and activated caspase3 measured by ELISA. The columns represent the fold-changes with standard errors of the mean (SEM) from 6 biological replicates (*p < 0.05; **p < 0.01; ***p < 0.001 - unpaired Student's *t*-test) that were normalised against GAPDH ELISA data (Panel **D**).

The expression of upstream regulators of CREB, namely ERK1/2, phospho-ERK1/2, and cAMP was measured by immunoblotting. A reduction of phospho-ERK1/2 but not that of total ERK1/2 level was observed at both radiation doses (Figure 3A and 3B). As the MAPK signalling pathway can be activated by PKA and PKC signalling, all proteins with a PKA- and PKC-motif were quantified using phospho-motif immunoblotting. PKC signalling was not affected by chronic radiation whereas PKA signalling was significantly inhibited at 6.0 Gy but not at 0.3 Gy (Figure 4A–4B). As PKA signalling is activated by cAMP levels, these were quantified. A reduced cAMP level was observed only at 6.0 Gy (Figure 4C and 4D) which is in good agreement with the reduced expression of the phosphorylated form of A-kinase anchoring protein Akap5. This protein anchors PKA kinase to cytoskeletal proteins and transfers the signal carried by cAMP to intracellular effectors [25].

### Chronic irradiation does not induce cell death or inhibit adult neurogenesis in hippocampus

**Figure 5:**
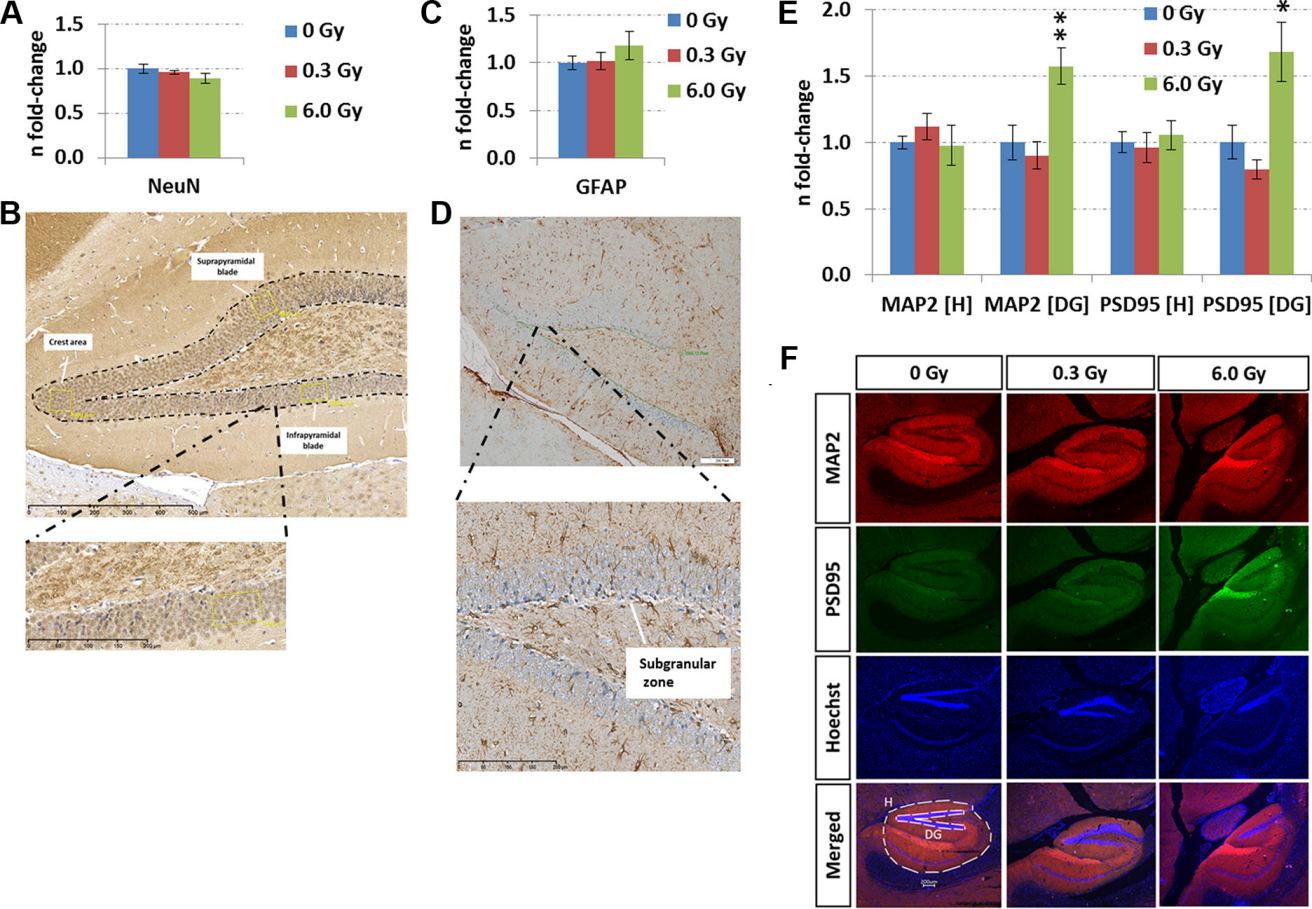
Analysis of adult neurogenesis and *in situ* quantification of MAP2 and PSD95 levels. Panels **A** and **C** show the fold-changes with standard errors of the mean (SEM) from NeuN and GFAP expression, respectively. The immunohistochemistry analysis was performed in a double-blinded fashion. Differences were considered to be significant when *p*-values were ≤ 0.05 using unpaired Student's *t*-test; *p < 0.05; **p < 0.01; ***p < 0.001. Panels **B** and **D** show representative images from NeuN and GFAP stainings, respectively. Immunohistochemical staining for NeuN was performed to assess the neuronal density in the granular cell layer (GC) of the dentate gyrus (DG). Counting was carried out in a rectangular field of 4,000 μm^2^in the suprapyramidal and infrapyramidal blade and in the crest area of the DG (yellow boxes). The number of positive cells in each of the areas was recorded separately, followed by statistical analysis of the mean from 4–6 biological replicates (*n* = 6: sham-irradiated; *n* = 4: irradiated). GFAP-expression in the subgranular zone (SGZ) was evaluated by counting immune-positive cells located at the border of the GC and hilus (HL, *n* = 6). The length of the borderline was measured and was used as normalisation for the number of positive cells for GFAP. Panels **E** and **F** show the data from sequential immunofluorescence from hippocampus (H) and dentate gyrus (DG) at the two radiation dose rates (doses). The columns represent the fold-changes with standard errors of the mean (SEM) from 6 biological replicates regarding MAP2 (red – microtubule-associated protein2), PSD95 (green – disks large homolog 4 [DLG4]), Hoechst and merged intensities within the hippocampal and DG region. The MAP2 / PSD95 intensity was normalised against nuclear Hoechst intensity in the region of interest. *p < 0.05; **p < 0.01; ***p < 0.001 (unpaired Student's *t*-test); magnification: 4×.

The evaluation of adult neurogenesis by counting Ki67^+^-cells (highly proliferating progenitor cells), GFAP^+^-cells (neural stem cells) in the subgranular zone, and NeuN^+^-cells (mature neurons) in the granular zone of the hippocampus revealed no alteration at either dose (dose rate) (Figure 5A–5D, Supplementary Figure S2). The stable number of mature neurons was consistent with unchanged level of apoptosis-inducing caspase-3 as evaluated by ELISA (Figure 4C and 4D) and *in situ* immunohistochemistry (Supplementary Figure S2). This suggests that the observed molecular changes in memory-related signalling pathways did not arise from a changed cellular process of adult neurogenesis or cell death in the hippocampus.

### Chronic irradiation increases synaptic proteins in the dentate gyrus

As the analysis of biological functions and diseases revealed a number of degenerative mechanism related to axons and membrane projections (Figure 2G), an immunofluorescence quantification of the post-synaptic density protein 95 (PSD95) and the microtubule-associated protein 2 (MAP2) was performed. Increased expression of both proteins in the dentate gyrus but not in the complete hippocampus was noted (Figures 5E and 5F). This correlated well with the mass spectrometry data showing that MAP2 and PSD95 were not significantly changed in the whole hippocampus at either radiation dose (fold-changes at 0.3 Gy/6.0 Gy: Map2 − 1.21/0.90; PSD95 (Dlg4) − 0.88/0.94) (Supplementary Table S1). Only phospho-MAP2 (Ser1791) expression was significantly downregulated at 6.0 Gy (fold-change: 0.5) (Supplementary Table S2).

### Chronic irradiation diminishes neuroinflammation and lipid peroxidation

**Figure 6:**
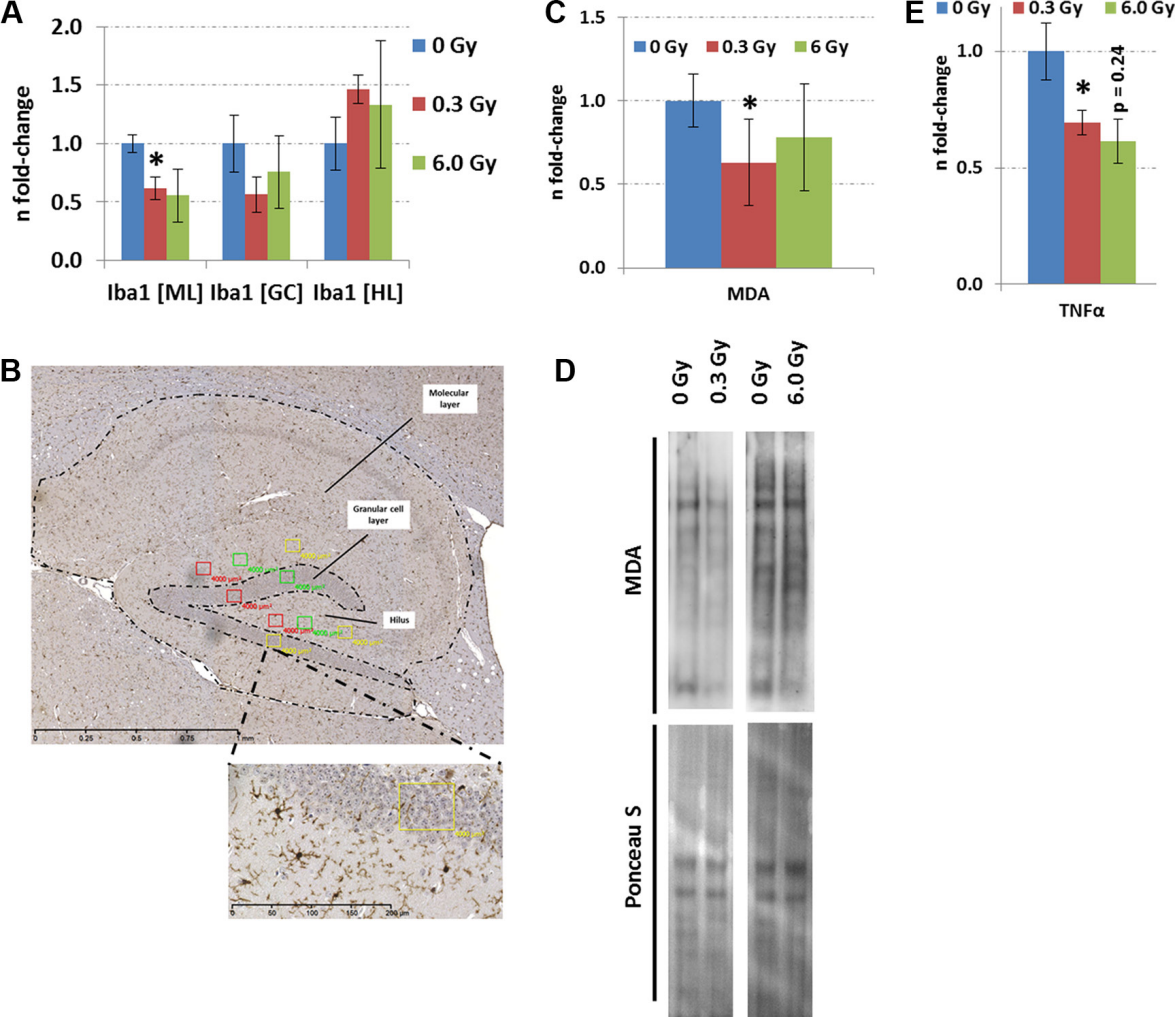
Analysis of neuroinflammation and lipid peroxidation in hippocampus after chronic irradiation. Panel **A**, **C** and **E** show the fold-changes with standard errors of the mean (SEM) from Iba1, MDA protein content and TNFα analysis. The immunohistochemistry analysis was performed in a double-blinded fashion. Differences were considered to be significant when *p*-values were ≤ 0.05 using unpaired Student's *t*-test; *p < 0.05; **p < 0.01; ***p < 0.001. Six biological replicates per group were used. Panel **B** shows a representative image from the Iba1 staining. The number of Iba1-positive cells was established by counting three rectangular fields of 4,000 μm^2^ in each biological replicate (*n* = 6) within the molecular layer (ML), granule cell layer (GC) and hilus (HL). The means were calculated from each cell region separately. Panel **D** shows the visualisation of proteins with MDA modification from a representative immunoblot. MDA; malondialdehyde.

The quantification of activated Iba1^+^-microglia, markers of neuroinflammation, demonstrated a reduction only in the molecular layer of the hippocampus at 0.3 Gy but not in the granular layer or hilus (Figure 6A and 6B). This was accompanied with a significantly reduced level of *TNFα* at this dose (Figure 6E). Moreover, a reduction in lipid peroxidation, evaluated by quantification of the total protein content modified with malondialdehyde (MDA) was observed (Figure 6C and 6D). At 6.0 Gy, no significant changes in these inflammation or oxidative stress markers were noted (Figure 6).

## DISCUSSION

ApoE knockout mice have been used as a model of AD as they exhibit mild neurodegenerative changes and behavioural abnormalities relevant to the early stages of this disorder, including synaptic and dendrite loss, lipid peroxidation, cellular stress, behavioural alterations in Morris water maze test and deficits in long-term potentiation (LTP) [26–30]. We used this mouse model to study whether chronic low-dose-rate radiation could be a potential risk factor in AD aetiology. This study shows a significant effect of the dose rate of 20 mGy/day with a cumulative dose of 6.0 Gy in 300 days on the phosphorylation status of the hippocampal proteome. Several proteins that showed a radiation-induced change in their phosphorylation status were associated with synaptic plasticity.

The data indicated a reduction in phosphorylation of the tau protein at site Ser554 at 6.0 Gy. This phosphorylation site has not been found before in the context of radiation biology and AD research, and its function remains unknown. It has been shown that a single dose of 0.5 Gy administered to neonatal NMRI mice leads to long-term cognitive dysfunction and increased level of total tau in the adult mouse brain [7]. Similarly, a heavy-ion dose of 0.1 Gy induced the formation of insoluble Aβ 6 months post-irradiation in AD mouse model [9]. These studies together with the data presented here suggest that ionising radiation may accelerate AD symptoms.

Even the dose rate of 1 mGy/day used in this study resulted in significant changes in the hippocampus that were distinct of those found at the dose rate of 20 mGy/day. An activation of Rac1 signalling was observed only at this very low dose rate. Activation of this pathway promotes actin depolymerisation and thus induces impairment in axonal outgrowth and elongation [31], especially if the level of inactive phospho-cofilin compared to total cofilin is low [20]. This, in combination with the observed changes in the phosphorylation status of neurofilament and actin- and microtubule-related proteins noted in the phospho-proteomics study, may inhibit synaptic plasticity as well as memory and learning.

CREB is an essential regulator of synaptic plasticity, neuroprotection and memory formation [32]. It is reduced in cognitive and neurodegenerative disorders such as AD [33] and is connected with the Rac1-Cofilin pathway [20]. We found an inhibition of CREB signalling in the chronically irradiated hippocampus. These changes correlated with the reduced cAMP levels, inhibition of PKA activity and reduced phosphorylation of MAPK upstream of CREB. It is known that molecular changes in Akap5 levels disrupt cAMP organisation and PKA signalling in the hippocampus and induce neurological disorders including AD [34]. Our global phosphoproteomics data indicated reduced phosphorylation of Akap5 at 6.0 Gy.

ARC is a CREB target protein, the expression of which has been shown to persistently decrease in the hippocampus of NMRI mice 7 months after a dose of 1.0 Gy [20]. Reduced levels of ARC have been shown to impair the maintenance of LTP and spatial memory consolidation [35]. Acute inhibition of ARC synthesis induced a loss of nascent F-actin at synaptic sites associated with dephosphorylation of cofilin [36]. Thus, ARC seems to be involved in the dephosphorylation of cofilin and, at least in part, may explain the imbalance between total and phospho-cofilin at 0.3 Gy found in this study.

This study shows that chronic low-dose-rate radiation resulted in anti-inflammatory and anti-oxidative effects in hippocampus, especially at 0.3 Gy. This was seen as a decreased number of Iba1-positive microglia, downregulation of TNFα expression and reduced lipid peroxidation. Increasing data suggest that this may be due to disturbances in MAPK signalling [37, 38], a pathway noted to be inhibited in our study.

The negative effect of chronic low-dose-rate radiation on lipid peroxidation observed here is not caused by changed levels of antioxidant proteins such as glutathione S-transferases (Gstm1, Gstp1), superoxide dismutases (Sod2, Sod1) or thioredoxins (Txn, Prdx3) as they were not found altered in the global proteomics analysis. The reduction in lipid peroxidation seen here is similar to that observed in long-term after moderate doses of high-dose-rate radiation [20, 39]. These previous studies suggested a possible association with inactivated mitochondria producing less reactive oxygen species (ROS) [23, 39]. Low-dose radiation may activate the oxidative stress defense system and reduce ROS-related injuries such as lipid peroxidation [40].

In contrast to this study, we and others have shown previously that high-dose-rate ionising radiation may stimulate the transformation of resting microglia to a reactive state that is associated with upregulation of pro-inflammatory cytokines such as TNFα [20, 41, 42]. Suppression of micgroglia-induced neuroinflammation is capable of attenuating AD symptoms and improving behavioral performance in a mouse model [43].

Due to the high importance of hippocampal neurogenesis for functional plasticity in the adult brain [13], the number of mature neurons, highly proliferating progenitors and neural stem cells was quantified. No significant difference in these numbers was found suggesting that adult neurogenesis is not affected by chronic low-dose-rate radiation. In agreement with this, no radiation-induced neuronal cell death (activated caspase-3) was observed. Recently, we demonstrated that a single low-dose high-dose-rate exposure to gamma-rays (0.1 Gy) at postnatal day 10 leads to a significant reduction in adult neurogenesis [20]. These data correlated well with the adverse consequences of single high radiation doses (> 5 Gy) for adult neurogenesis as reviewed recently [13]. We suggest that the distinct outcomes of these studies on neurogenesis may be related to the dose rate or age at exposure.

Importantly, we show here that chronic irradiation induces the expression of the synaptic proteins MAP2 and PSD95 only in the dentate gyrus but not in the complete hippocampus (6.0 Gy). This is in agreement with previous studies using high-dose-rate radiation [20, 44]. An increase in hippocampal MAP2 levels may be a compensatory response to the age-related functional decline of brain function in C57BL/6J mice [45]. This may indicate that chronic low-dose-rate radiation targets the integration process of newborn neurons in existing synaptic wires and inhibits in that way synaptic plasticity.

## CONCLUSIONS

This study is the first of its kind to elucidate the protein modification alterations in hippocampus when exposed to chronic low-dose-rate ionising radiation. Although it cannot distinguish between the effects of dose and dose rate, the comparison with previous high-dose-rate studies indicate that both play a role in the observed alterations. Furthermore, chronic irradiation seems to result in a unique molecular fingerprint, highlighting an overwhelming role of phosphorylation events of hippocampal proteins. These data are especially important considering that an increasing number of people are exposed to chronic radiation either in occupational or medical situations but more research in the area of molecular epidemiology is needed to validate these first observations.

## MATERIALS AND METHODS

### Ethics statement, irradiation of animals and tissue collection

Experiments were carried out in accordance to Japanese ethics committee (processing numbers: 24–20, 24–21 and 25–16). All experiments were conducted according to the legal regulations in Japan.

Female C57Bl/6 mice (8 weeks old) with Apoe^−/−^ deletion (specific-pathogen-free B6.129P2-ApoE^tm1Unc/J^, Charles River) were chronically irradiated with 1 mGy/day or 20 mGy/day (^137^Cs, gamma-rays) over 300 days (cumulative dose: 0.3 Gy and 6.0 Gy, respectively) at the Department of Radiobiology, Institute for Environmental Sciences, Aomori, Japan. Dosimetry was carried out with two ionisation chambers and TLDs [46]. The mice were maintained throughout the experiment in a specific-pathogen-free (SPF) facility. Radiation exposure was continuous for 22 h per day. The remaining 2 h were used to maintain the rooms and cages with animals. Animals were sacrificed via CO_2_ asphyxiation after 300 days of chronic low-dose-rate irradiation. Brains were excised and transferred to ice-cold phosphate buffered saline (PBS), rinsed, and dissected under stereomicroscopic inspection in cold. Hippocampi from each hemisphere were separately sampled, gently rinsed in ice-cold PBS and snap-frozen in liquid nitrogen for protein and RNA studies. For immunohistochemistry and immunofluorescence studies, one hemisphere of the brain was immediately fixed by 4% buffered formalin and kept for 2–3 days light protected at room temperature (RT).

### Isolation of total protein and RNA

The frozen hippocampi were homogenised in 6 M urea, 2 M thiourea, 10 mM DTT, 20 mM TEAB, pH 7.5, containing protease and phosphatase inhibitors (cOmplete Protease Inhibitor and PhosSTOP, Roche Diagnostics) manually using a mortar on ice. Homogenates were briefly vortexed and sonicated. The samples were stored at −20°C before further use.

Total RNA from frozen hippocampi was isolated and purified by mirVana^™^ Isolation Kit (Ambion) according to the manufacturer's instructions. Total RNA was eluted with nuclease-free water. The optical density (OD) ratio of 260/280 was measured using Nanodrop spectrophotometer (PeqLab Biotechnology); it ranged between 1.9 and 2.1. Eluates were stored at −20°C until further analysis.

In total, 18 mice per condition were used for analysis consisting of 6 biological replicates for immunoblotting or ELISA, 6 for mass spectrometry-based proteome analysis, 3 for RNA experiments and 6 for immunohistochemistry and immunofluorescence. Samples for RNA and immunohistochemistry and immunofluorescence originated from same mice.

### Mass spectrometry-based proteome analysis

#### Sample processing

**Figure 7:**
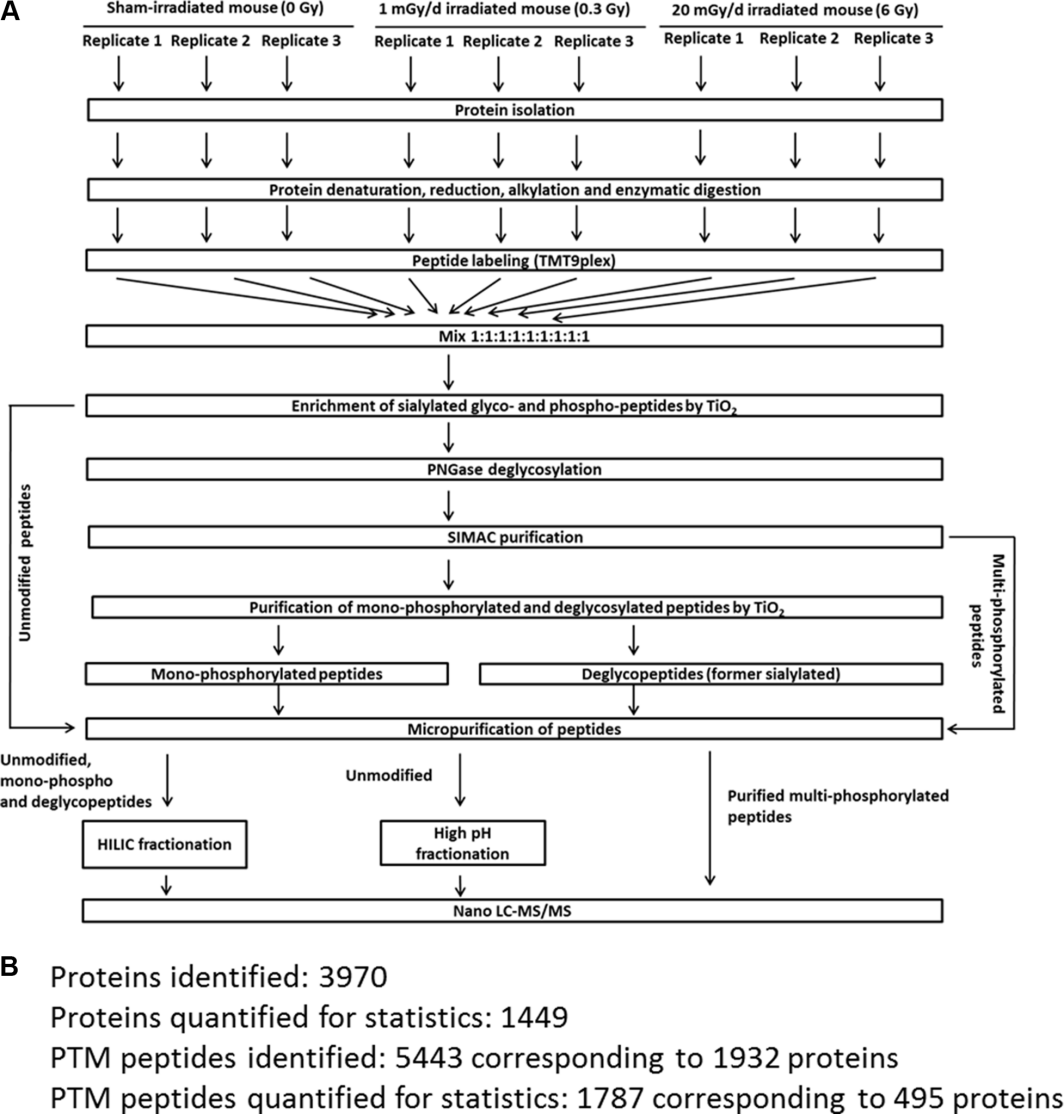
Proteomics workflow used in this study. Panel **A** shows the workflow to analyse phosphorylated, N-linked sialylated glycoproteins and unmodified proteins using mass spectrometry. Panel **B** shows the number of PTM and protein identifications as well as the numbers used for final quantification. The criteria for protein and PTM quantification including statistics are described in the Material and Methods section. PTM; post-translational modification.

The workflow and the number of identifications and quantifications of the proteomics experiments are shown in Figure 7. The mass spectrometry-based proteome analysis was performed as described previously [24]. Briefly, 100 μg tryptic peptides per brain region and condition were labelled by a TMT-9plex approach (AB Sciex) that was performed as follows: TMT-126/−127N/127C was used for sham-irradiated samples, TMT-128N/−128C/−129N for 0.3 Gy-irradiated samples and TMT-129C/−130N/−130C for 6 Gy-irradiated samples. Six biological replicates were used per condition. The labelling was performed according to manufacturer's instructions.

Multi- and monophosphorylated peptides and sialylated N-linked glycopeptides were separated from unmodified peptides using a TiO_2_-SIMAC-TiO_2_ (TiSH) workflow [47–50]. Following SIMAC (sequential elution from IMAC beads) [51], multiphosphorylated peptides were enriched and eluted separately from the monophosphorylated and deglycopeptides that were, in turn, separated in a second TiO_2_ step (Figure 7A). The eluted modified peptides from the first TiO_2_ step were deglycosylated to remove N-linked glycans [48]. Hydrophilic interaction chromatography (HILIC) was used as sample fractionation prior to nano liquid chromatography-tandem mass spectrometry (LC-MS/MS).

High pH fractionation was used to increase protein identification of the unmodified peptides. Combined labelled samples (100 μg) containing unmodified peptides were dissolved in 50 mM TEAB, pH 8.5, and loaded on a R3 micro-column that was equilibrated with 50 mM TEAB, pH 8.5. The column was washed with 50 μl 50 mM TEAB, pH 8.5, followed by a wash with 50 μl water. The peptides were sequentially eluted by 30 μl 7%, 10%, 12%, 15%, 17.5%, 22% and 60% acetonitrile (ACN). The eluates were dried and analysed by mass spectrometry.

### Reversed-phase nano-LC-ESI-MS/MS

The peptides resuspended in 0.1% formic acid (FA) were automatically injected and loaded on a ReproSil-Pur C18 AQ (Dr. Maisch, Ammerbuch-Entringen, Germany) in-house packed trap column (2 cm × 100 μm; 5 μm). The peptides were separated at 250 nl/min on an analytical ReproSil-Pur C18 AQ (Dr. Maisch, Ammerbuch-Entringen, Germany) packed in-house column (17 cm × 75 μm; 3 μm) by reversed phase chromatography which was operated on an EASY-nanoLC system (Thermo Fisher Scientific, Odense, Denmark). Mobile phase was set to 95% ACN/ 0.1% FA (B) and water/0.1% FA (A). The gradient was from 1% to 34% solvent B in 80 min (unmodified peptides), 40 min (mono-phosphoralyated- and deglycosylated-peptides) or 110 min (multiphosphorylated peptides), 34–50% B in 7 min, 50–100% B in 5 min and 8 min at 100% B. The nano-LC was online connected to an Orbitrap Fusion (Thermo Fisher Scientific; only high-pH fractionated unmodified peptide samples) or Q Exactive HF Hybrid Quadrupole-Orbitrap mass spectrometer (Thermo Fisher Scientific; all other samples) operating in positive ion mode and using data-dependent acquisition. The Orbitrap acquired the full MS scan with an automatic gain control of a target value of 1 × 10^6^ ions and a maximum fill time of 120 ms. Each MS scan was acquired at high-resolution (60,000 full-width half maximum (FWHM)) at m/z 200 in the Orbitrap with a mass range of 400–1400 Da. The 12 most abundant peptide ions were selected from the MS for HCD fragmentation (collision energy: 34 V) if they were at least doubly charged. Fragmentation was performed at high resolution (60,000 FWHM) for a target of 1 × 10^5^ and a maximum injection time of 60 ms using an isolation window of 1.2 m/z and a dynamic exclusion of 20 s.

### Data analysis

Raw data were searched against the SwissProt database and UniProt mouse reference database via Mascot (v2.3.02, Matrix Science) and Sequest HT search engines, respectively, using Proteome Discoverer (v1.4.1.14, Thermo Fisher Scientific). A precursor mass tolerance of 10 ppm and a product ion mass tolerance of 0.02 Da were applied allowing not more than one missed cleavage for trypsin. Fixed modifications included carbamidomethylation of Cys and TMT9-plex labeling for Lys and N-terminal. Variable modifications contained phosphorylation on Ser/Thr/Tyr and deamidation of Asn. The TMT datasets were quantified using the centroid peak intensity with the “reporter ions quantifier” node. To ensure a high-confident identification of peptides, we used the Mascot percolator algorithm (*q* value filter set to 0.01), Mascot and Sequest HT peptide rank 1 and a cut-off value of Mascot score ≥ 22 as well as Sequest HT ΔCn of 0.1. Moreover, a cut-off value of Xcorr score for charge states of +1, +2, +3 and +4 higher than 1.5, 2, 2.25 and 2.5, respectively, were considered for further analysis. Subsequently, these peptides were filtered against a Decoy database resulting into a false discovery rate (FDR) of lower than 0.01 (FDR < 0.01). PhosphoRS was used to localise phosphorylation sites with a confidence filter of 99%. Six biological replicates without missing values were considered for the statistical analysis. Quantification was performed on the log2-values of the measured peptide intensities and the data were normalised based on the median. Modified peptides were merged with the R Rollup function (http://omics.pnl.gov/software/danter) allowing for one-hit-wonders and using the mean of the normalised intensities for each peptide. Quantification of proteins was obtained by merging the unmodified peptides with the R Rollup function considering at least 2 unique peptides not allowing for one-hit-wonders and using the mean of the intensities. Subsequently, the mean over the experimental conditions for each peptide in each replicate was subtracted in order to merge the data from different replicates. Proteins, phosphopeptides and formerly sialylated N-linked glycopeptides with a consensus motif for N-linked glycosylation (NXS/T/C; where X # P) were considered to be significantly deregulated if they were identified by at least two unique peptides (proteins) or at least one unique peptide (post-translationally modified peptides) in 6 biological replicates without missing values. Phosphorylated and deglycosylated peptides were normalised based on the protein expression in each of the replicates including the proteins with two unique peptides to ensure that deregulation occurred on PTM level and not on protein level. Significant up/down-regulations between experimental conditions were calculated allowing a FDR of 0.05 without missing values. We applied combined limma and rank product tests [52], subsequently corrected for multiple testing according to Storey.

The mass spectrometry proteomics data was deposited to the ProteomeXchange Consortium [53] via the PRIDE partner repository with the dataset identifier PXD003969 (Username: reviewer76492@ebi.ac.uk; Password: 2XqkmUme).

### Bioinformatics analysis

Deregulated proteins were assigned to molecular functional classes using PANTHER classification system software (http://www.pantherdb.org) and the general annotation from UniProt (www.uniprot.org). To identify radiation-affected signalling pathways, a signalling pathway analysis was performed with all altered proteins for each dose group using INGENUITY Pathway Analysis (IPA) (http://www.ingenuity.com) applying databases of experimental and predictive origin. Furthermore, IPA was also used to visualise significantly (z-score ≥ 2: activation or z-score ≤ −2: inhibition; *p*-value ≤ 0.05) changed biological functions / diseases by the z-scores and *p*-values derived from the Fisher's exact test across all observations.

The ClueGO plug-in of Cytoscape (http://apps.cytoscape.org/apps/cluego) was used for cellular compartment gene ontology (GO) term annotation. Only significantly enriched terms with a *p*-value ≤ 0.05 were reported, subsequently corrected for multiple testing according to Benjamini & Hochberg.

### Quantification of proteins and phosphorylation-motifs by immunoblotting

Hippocampal protein extracts (15 μg) were separated on 12% SDS polyacrylamide gels and transferred to nitrocellulose membranes (GE Healthcare) via BIO-RAD Criterion^TM^ Blotter system. The membranes were blocked with Roti^R^-Block solution (Roth), washed and incubated overnight at 4°C with primary antibody dilutions as recommended by the manufacturer: GAPDH – sc-47724, Santa Cruz; Rac1 – ab33186, Abcam; cofilin – 3312, Cell Signalling; p-Cofilin (Ser3) – 3311, Cell Signalling; CREB – 4820, Cell Signaling; phospho-CREB (Ser133) – 9198, clone 87G3, Cell Signaling; ARC – A8344, Sigma Aldrich; p44/42 MAPK (ERK 1/2) – 9102, Cell signalling; phospho-p44/42 MAPK (ERK 1/2) (Thr202/Tyr204) – 9101, Cell Signaling; phospho-PKC Substrate Motif (R/KXpSX(R/K) MultiMab Rabbit Monoclonal Antibody – 6967, Cell Signalling; phospho-PKA Substrate (RRXS*/T*) (100G7E) Rabbit Monoclonal Antibody – 9624, Cell Signalling; and goat polyclonal antibody against malondialdehyde (MDA)-tagged proteins (HRP-linked) - ab20703, Abcam. Following secondary antibodies were used: rabbit anti-mouse IgG (HRP-linked, ab6728, Abcam) and goat anti-rabbit IgG (HRP-linked, ab6721, Abcam). The blots were incubated with appropriate horseradish peroxidase-conjugated secondary antibody in 8% milk for 1 h at RT and developed using ECL system (GE Healthcare) using standard protocol from the manufacturer. GAPDH was not significantly changed based on the global proteomics results in any sample and was therefore used as the loading control. Immunoblots were quantified with TotalLab TL100 software (www.totallab.com) using software-suggested background correction. Six biological replicates were used for statistical analysis (unpaired Student's *t*-test) with a significance threshold of 0.05.

### Enzyme-linked immunosorbent assay (ELISA)

A total of 5 μg of protein lysates were used to quantify Caspase3 (SEA626Mu, Cloud-Clone Corp.), TNFα (BMS607HS, eBioscience) and cAMP (KGE012B, R&D Systems) using manufacturer's instructions. The plates were measured on a FLUOstar Omega (BMG Labtech) at the recommended wavelength of the manufacturer. All assays were normalised against an ELISA against GAPDH (ab176642, Abcam). GAPDH was not changed in the proteomics and transcriptomics data and was therefore used for data normalisation. Six biological replicates were analysed in duplicates. The mean of each technical triplicate was normalised against the mean of the representative GAPDH technical replicates. Statistical analysis was performed via unpaired Student's *t*-test and the data are presented as fold-changes with the standard error of the mean (SEM).

### Pathway-focussed gene expression analysis related to synaptic plasticity

Sham- and 6.0 Gy-irradiated hippocampal RNA isolates (100 ng) were used to quantify the gene expression of 84 mRNA transcripts related to synaptic plasticity (RT2 Profiler Mouse Synaptic Plasticity – PAMM-126Z, Qiagen). The relative expression of each mRNA was normalised against the median of all 84 target genes using the equation 2^−ΔΔCt^, where ΔΔCt = ΔCt_irradiated_ – ΔCt_sham_ and ΔCt = Ct_target-mRNA_ – Ct_median-of-84-target-genes_. Three biological replicates were used within each group. Gene expression changes were considered significant if they reached a *p*-value of ≤ 0.05 and if they had a fold-change of ≥ 1.2 or ≤ −1.2. The threshold of ± 1.2 was based on the average experimental technical variance (8.4%) and biological variance (6.9%) of a set of 14 overlapping target genes as reported elsewhere [54]. Three biological replicates were used.

### Immunohistochemistry/Immunofluorescence

Formalin-fixed and paraffin-embedded tissues were prepared and processed using standard techniques [55]. One μm thick single sagittal brain sections were dewaxed, rehydrated and heated in citrate buffer (pH 6.0) for 30 min for evaluation of GFAP, Ki67, activated caspase-3, Iba1 and NeuN. Four μm thick sections were cut and stained with haematoxylin and eosin (H&E) followed by visual inspection for morphological aberrations. Quenching of endogenous peroxidase was performed with 3% H_2_O_2_ in methanol (v/v) for 20 min. Brain sections were incubated with primary antibody dilutions as recommended by the manufacturer. Following antibodies were used for immunohistochemistry: mouse monoclonal antibody against Ki67 (clone B56, 556003, BD PharMingen, Heidelberg, Germany, 1:100), mouse monoclonal antibody against NeuN (clone A60, MAB377, Merck Millipore, Schwalbach, Germany, 1:20), rabbit polyclonal antibody against Iba1, rabbit monoclonal antibody against cleaved caspase 3 (Asp175, #9661, Cell Signaling, Massachusetts, USA, 1:200) and rabbit polyclonal antibody against GFAP (Z0334, DakoCytomation, Hamburg, Germany, 1:350).

After application of the specific primary antibody, the slides were processed with the automated staining system i6000 (Biogenex, USA) according to manufacturer's instructions, including the incubation with the appropriate secondary antibody (MoMap Kit 760-137, Ventana, Tucson, AZ, USA) according to manufacturer's instructions or biotinylated using goat anti-rabbit IgG BA-1000, Vector Laboratories, Burlingame, CA, USA, 1:750). Haematoxylin was used as a counterstain and diaminobenzidine (DAB) was used for the immunohistochemical stain. Visualisation was performed using a streptavidin–horseradish peroxidase system (HK330-9K; Biogenex, San Ramon, CA, USA). After dehydration, the slides were air-dried and mounted with Eukitt^®^ (Sigma-Aldrich, Germany) and cover-slipped. Each immunohistochemical staining was performed simultaneously with identical incubation times and concentrations for the primary and secondary antibody and DAB solution.

Light microscopic evaluation of the staining was performed on the stained sagittal sections encompassing all relevant brain structures. Images were taken with the Olympus BX43F microscope equipped with DP25 digital camera (Olympus Deutschland GmbH, Hamburg) (GFAP stainings) and Hamamatsu NanoZoomer 2.HT^®^ Slide scanning system (Hamamatsu Photonics K.K., Japan) (other stainings). All images were analysed using identical software settings by two qualified pathologists experienced in mouse histopathology (FN, DJ).

The area of granule cell layer (GC) and subgranular zone (SGZ) of the hippocampal dentate gyrus (DG) was measured using imaging software NDP.view (Hamamatsu Photonics K.K., Japan). Subsequently, the number of Ki67-positive cells in the GC and SGZ were counted (*n* = 6). The number of Iba1-positive cells was established by counting three rectangular fields of 4,000 μm^2^ in each biological replicate (*n* = 6) within the molecular layer [52], GC and hilus (HL). The means were calculated from each cell region separately. Immunohistochemical staining for NeuN was performed to assess the neuronal density in the GC of the DG. Counting was carried out in a rectangular field of 4,000 μm^2^ in the suprapyramidal, infrapyramidal blade and in the crest area of the DG. The number of positive cells in each of the areas was recorded separately, followed by statistical analysis of the mean from 4–6 biological replicates (*n* = 6 for sham-irradiated; *n* = 4 for irradiated). To detect cleaved caspase 3 immunoreactivity, the whole hippocampal formation was examined via light microscopy (*n* = 6). GFAP-expression in the SGZ was evaluated by counting immunopositive cells located at the border of the GC and HL (*n* = 6). The length of the borderline was measured with the labSens imaging software (Olympus Deutschland GmbH, Hamburg) and the number of positive cells for GFAP was normalised against the length. The complete immunohistochemistry analysis was performed in a double-blinded fashion. Differences were considered to be significant when *p*-values were ≤ 0.05 using unpaired Student's *t*-test.

For immunofluorescence, auto-fluorescence was blocked by 0.1% Sudan black in 70% ethanol after epitope retrieval in citrate buffer. After a goat serum block, slides were incubated overnight with rabbit anti-mouse primary antibody against MAP-2 (ab32454–Abcam, Germany) followed by goat anti-rabbit Cy3-Fab-fragment IgG secondary antibody (111-167-003 - Jackson ImmunoResearch, UK) using manufacturer's instructions. Subsequently, the slides were washed in PBS and incubated overnight with rabbit anti-mouse primary antibody against PSD-95 (ab18258–Abcam, Germany), followed by goat anti-rabbit Alexa-fluor IgG secondary antibody (111-545-144 - Jackson ImmunoResearch, UK) using manufacturer's indications. The slides were nuclear stained with Hoechst and mounted with antifade fluorescence mounting media. Sample processing was done under identical conditions on the same day. All steps were performed in a humid chamber in the dark. All images were analysed using identical software settings. The MAP-2 / PSD-95 intensity in the region of interest was normalised against the Hoechst intensity within this region. Six biological replicates were used in all cases. Statistical significance was calculated with unpaired Student's *t*-test using *n* = 6. Respective controls of specificity of this sequential immunofluorescence approach are reported elsewhere [20].

## SUPPLEMENTARY MATERIALS FIGURES AND TABLES





## Abbreviations

ACN: acetonitrile
AD: Alzheimer's disease
CT: computer tomography
DG: dentate gyrus
ELISA: enzyme-linked immusorbent assay
FWHM: full-width half maximum
FDR: false discovery rate
GO: gene ontology
GC: granule cell layer
HL: hilus
HILIC: hydrophilic interaction chromatography
H: hippocampus
IAA: iodacetamide
IPA: Ingenuity pathway analysis
LTP: long-term potentiation
LC-MS/MS: liquid chromatography-tandem mass spectrometry
MDA: malondialdehyde
ML: molecular layer
OD: optical density
PBS: phosphate buffered saline
PTM: post-translational modification
Q: quartile
RT: room temperature
ROS: reactive oxygen species
SIMAC: sequential elution from IMAC beads
SEM: standard error of the mean
SPF: specific-pathogen-free
SGZ: subgranular zone
TiSH: TiO_2_-SIMAC-TiO_2_
