# Supplementary material for: Chronic low-dose-rate ionising radiation affects the hippocampal phosphoproteome in the ApoE−/− Alzheimer's mouse model
Source: Oncotarget. 2016 Sep 30;7(44):71817–32. doi: 10.18632/oncotarget.12376 (PMC5342125; doi:10.18632/oncotarget.12376)
Supplement: Supplementary file 1 [file oncotarget-07-71817-s001.pdf]

Chronic low-dose-rate ionising radiation affects the hippocampal phosphoproteome in the ApoE$^{-/-}$ alzheimer mouse model

Supplementary Materials

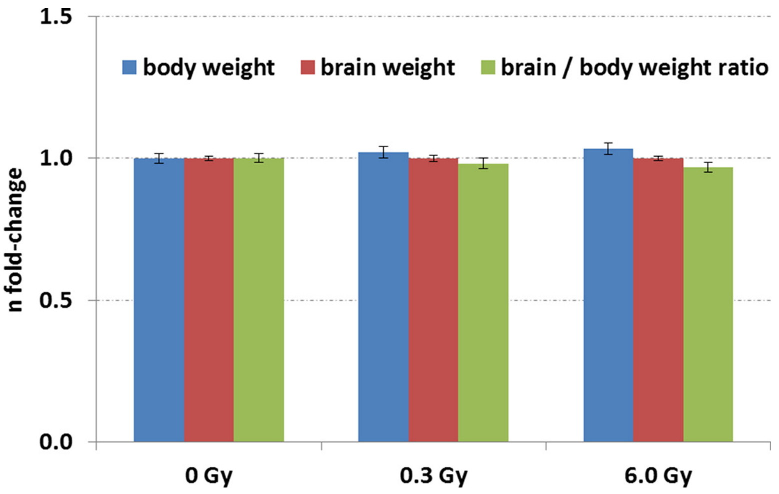

Supplemetary Figure S1: Body and brain weights in mice after chronic irradiation. Data of body and brain weights and the ratios of these are shown as fold-changes ± SEM (standard error of the means) from 16 biological replicates (Student´s *t*-test, unpaired).

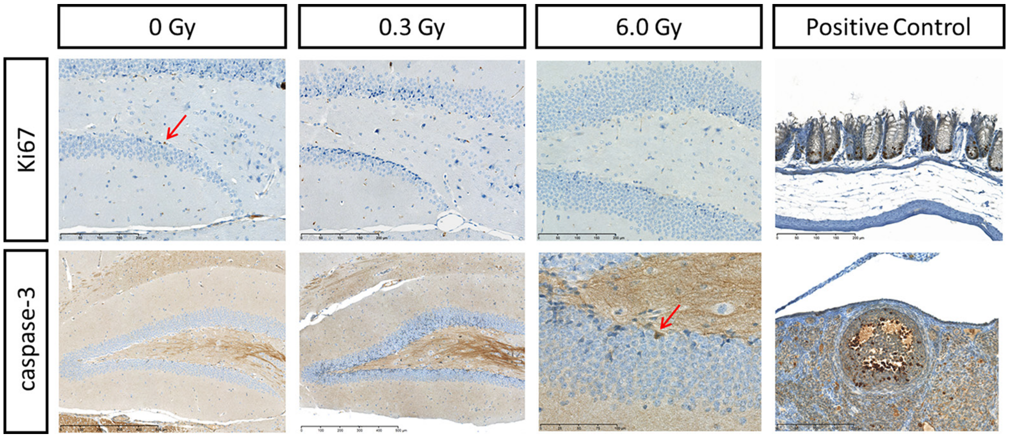

Supplemetary Figure S2: Immunohistochemistry of Ki67 and cleaved caspase-3. Representative images from sham-irradiated (0 Gy) and chronically-irradiated (0.3 Gy and 6.0 Gy) brain slides stained against Ki67 (n = 6) and activated caspase-3 (n = 6) are shown. Positive controls: Ki67 – murine colon with epithelial cells; cleaved caspase-3 – murine ovary with degenerated follicle. The stainings showed only randomised hits (arrows).

Supplementary Table S1: Protein list of quantifiable unmodified proteins and global standard deviation calculation within all groups (0 Gy, 0.3 Gy and 6.0 Gy). See Supplementary_Table S1

Supplementary Table S2: Significantly deregulated phosphopeptides and N-linked sialylated glycopeptides. See Supplementary_Table S2

Supplementary Table S3: Quantifiable phosphopeptides and N-linked sialylated glycopeptides. See Supplementary_Table S3

Supplementary Table S4: Targeted transcriptomics analysis of genes related to "Synaptic Plasticity". See Supplementary_Table S4

Supplementary Table S5: Visualisation of deregulated signalling pathways obtained using IPA software. The images are adapted from Qiagen without further modification. See Supplementary_Table S5
